# TR4 nuclear receptor increases prostate cancer invasion *via* decreasing the miR-373-3p expression to alter TGFβR2/p-Smad3 signals

**DOI:** 10.18632/oncotarget.3778

**Published:** 2015-04-27

**Authors:** Xiaofu Qiu, Jin Zhu, Yin Sun, Kun Fan, Dong-Rong Yang, Gonghui Li, Guosheng Yang, Chawnshang Chang

**Affiliations:** ^1^ Department of Urology, Guangdong No. 2 Provincial People's Hospital, Guangzhou, China; ^2^ George Whipple Lab for Cancer Research, Departments of Pathology, Urology, Radiation Oncology, and The Wilmot Cancer Center, University of Rochester Medical Center, Rochester, NY, USA; ^3^ Department of Urology, the Second Affiliated Hospital of Soochow University, Suzhou, China; ^4^ Chawnshang Chang Liver Cancer Center, Department of Urology, Sir Run-Run Shaw Hospital, Zhejiang University, Hangzhou, China; ^5^ Sex Hormone Research Center, China Medical University/Hospital, Taichung, Taiwan

**Keywords:** TR4, prostate cancer, metastasis, miR-373-3p

## Abstract

Testicular nuclear receptor 4 (TR4), a member of the nuclear receptor superfamily, may play important roles to modulate the metabolic diseases and prostate tumorigenesis. Here we found TR4 could increase prostate cancer (PCa) cell invasion. Mechanism dissection revealed that TR4 might increase PCa cell invasion *via* decreasing the miR-373-3p expression that resulted in the activation of the TGFβR2/p-Smad3 signals. The *in vivo* mouse model using orthotopically xenografted CWR22Rv1 cell line transfected with luciferase-reporter confirmed *in vitro* cell line studies showing TR4 increased PCa metastasis *via* decreasing the miR-373-3p expression. Together, these data suggest that TR4 may increase PCa metastasis *via* a newly identified signal and targeting these TR4/miR-473-3p/TGFβR2/p-Smad3 signals using TR4 antagonist or TR4-siRNA or miR-373-3p may allow us to develop a new potential therapeutic approach to better suppress PCa metastasis.

## INTRODUCTION

Prostate cancer (PCa) has the second highest mortality in men [1]. Most patients eventually relapse with recurrent metastatic PCa that has become castration resistant after androgen deprivation therapy (ADT) [2–4]. Identifying molecules and signals linked to enhanced PCa metastasis and development of new therapies to suppress the metastatic PCa may help us to better inhibit the PCa at the later castration resistant stage.

The testicular nuclear receptor 4 (TR4) is a transcriptional regulator that belongs to the nuclear receptor superfamily [5–8]. *In vivo* mouse studies suggested that TR4 might play important roles to modulate the progression of several diseases including metabolic disorders and various tumors [9–11]. Early studies revealed that TR4 might play a protective role to inhibit the prostate tumorigenesis and knocking-out TR4 in a mouse model (TR4KO) might increase PIN and/or prostatic carcinoma formation [12]. The role of TR4 in PCa metastasis, especially involving the regulation of microRNAs (miRNAs), however, remains to be further elucidated.

TGFβ/Smad3 signals play a critical role in the regulation of tumor progression including metastasis [13]. Interestingly, depending on different cellular contexts, TGFβ might either promote or suppress tumor progression [14], and TGFβ receptor II (TGFβR2) tranduces TGFβ signaling. miRNAs are small (< 22 nt), non-coding RNA molecules that bind to the 3′ untranslated region (3′ UTR) of their target mRNAs, to regulate gene expression at a post-transcriptional level [15]. More than 1,400 human miRNA sequences have been identified thus far and many of them have been linked to the cancer pathogenesis, including tumor initiation, proliferation and invasion [16]. Importantly, Walter et al. reported that differential profiles of miRNAs might play different roles that are linked to the aggressive behavior of PCa progression [17].

In this study, we found TR4 might be able to function through suppression of the miR-373-3p expression to alter the TGFβR2/p-Smad3 signals to enhance the PCa cell invasion.

## RESULTS

### TR4 increases PCa cell invasion

**Figure 1:**
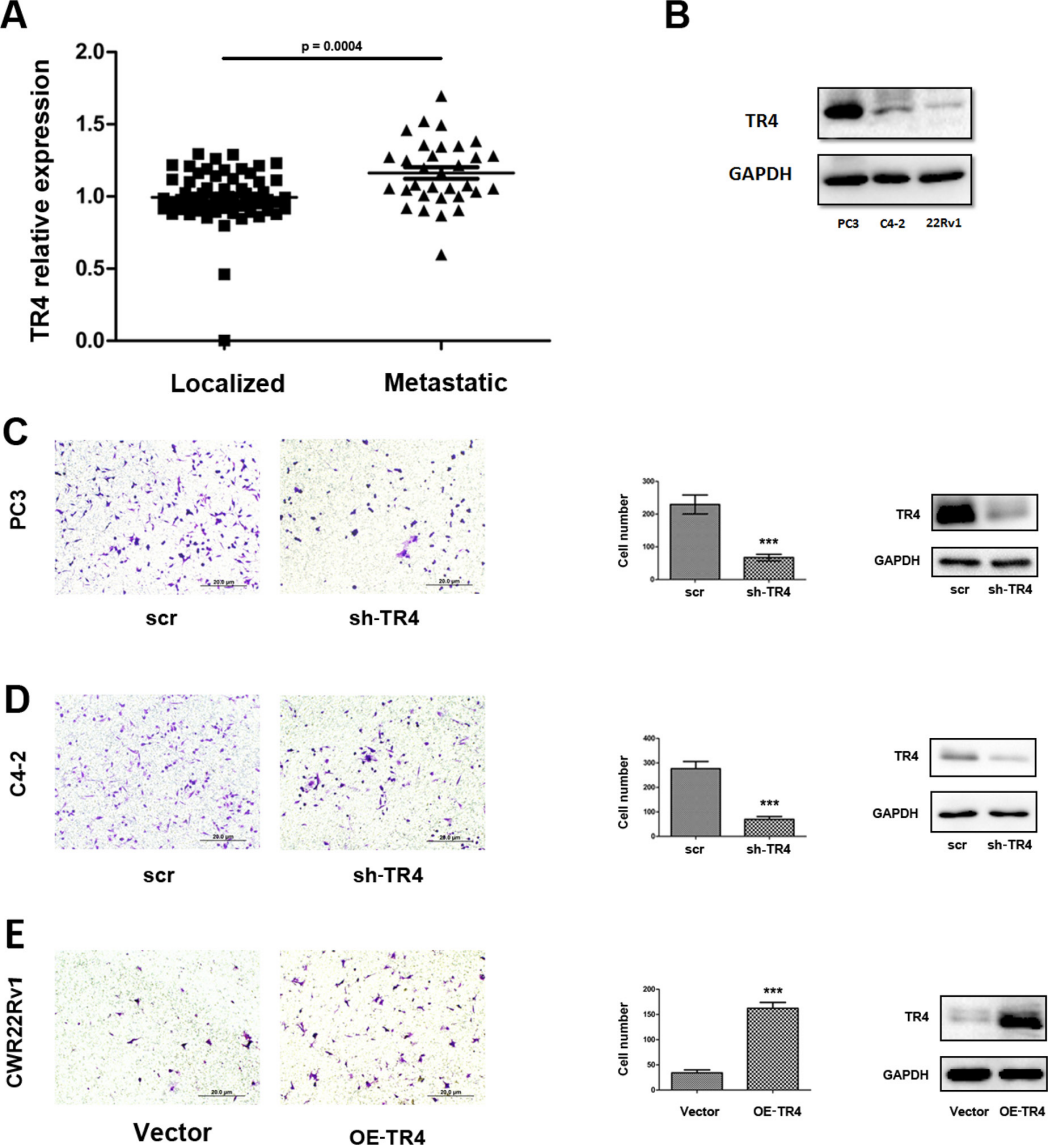
Effect of TR4 on PCa cell invasion **A.** GEO databases to analyze the PCa sample array with TR4 expression revealed that PCa tissues (*n* = 32). **B.** The different expression of TR4 in three PCa cell lines. In PC3 cells, the expression is highest and in CWR22Rv1 (22Rv1) cells, it is the lowest. **C–E.** After 48 hrs lentivirus transfection, cell were sded in cell invasion chambers with the inner wells coated with Matrigel and incubated for 24–36 hrs. Left panels: Representative microphotographs of invaded cells (100 ×). Middle panels: Quantitive analysis (cell numbers were counted in six randomly chosen microscopic fields per membrane). ***p < 0.001 Right panels: Western blot analysis for the knockdown or overexpression of TR4. **C–D.** Invasion assays were performed in C4–2 **(C)** or PC3 **(D)** cells transfected with sh-TR4 or scramble control. **E.** Invasion assays were performed in CWR22Rv1 cells transfected with overexpressed TR4 (OE-TR4) and vector control.

An early study [18] indicated the higher TR4 expression in tumor tissues of PCa patients with Gleason score 5 + 4 compared with those patients with Gleason score 3 + 3. Interestingly, using NCBI GEO databases [19] to analyze the PCa sample array with TR4 expression, we found that PCa metastatic tumors have a slightly higher TR4 expression than PCa localized tumors (*p* < 0.001) (Figure 1A).

We then applied 3 PCa cell lines, including C4–2, PC3 and CWR22Rv1, to confirm this clinical finding, and results revealed that TR4 was differentially expressed in these PCa cell lines with higher expression in PC3 and lower expression in CWR22Rv1 cells (Figure 1B). Importantly, using matrigel coated transwell invasion assays with TR4-shRNA to knock down TR4 in PC3 cells, we found that reduced TR4 decreased PCa cell invasion (Figure 1C). Similar results were also obtained when we replaced PC3 cells with C4–2 cells (Figure 1D). We also applied an opposite approach with addition of functional TR4-cDNA into CWR22Rv1 cells, and results revealed that increased TR4 significantly increased PCa cell invasion (Figure 1E).

Together, results from Figure 1A–1E proved TR4 might play positive roles to increase the PCa cell invasion.

### TR4 decreases miR-373-3p expression in PCa cells

**Figure 2:**
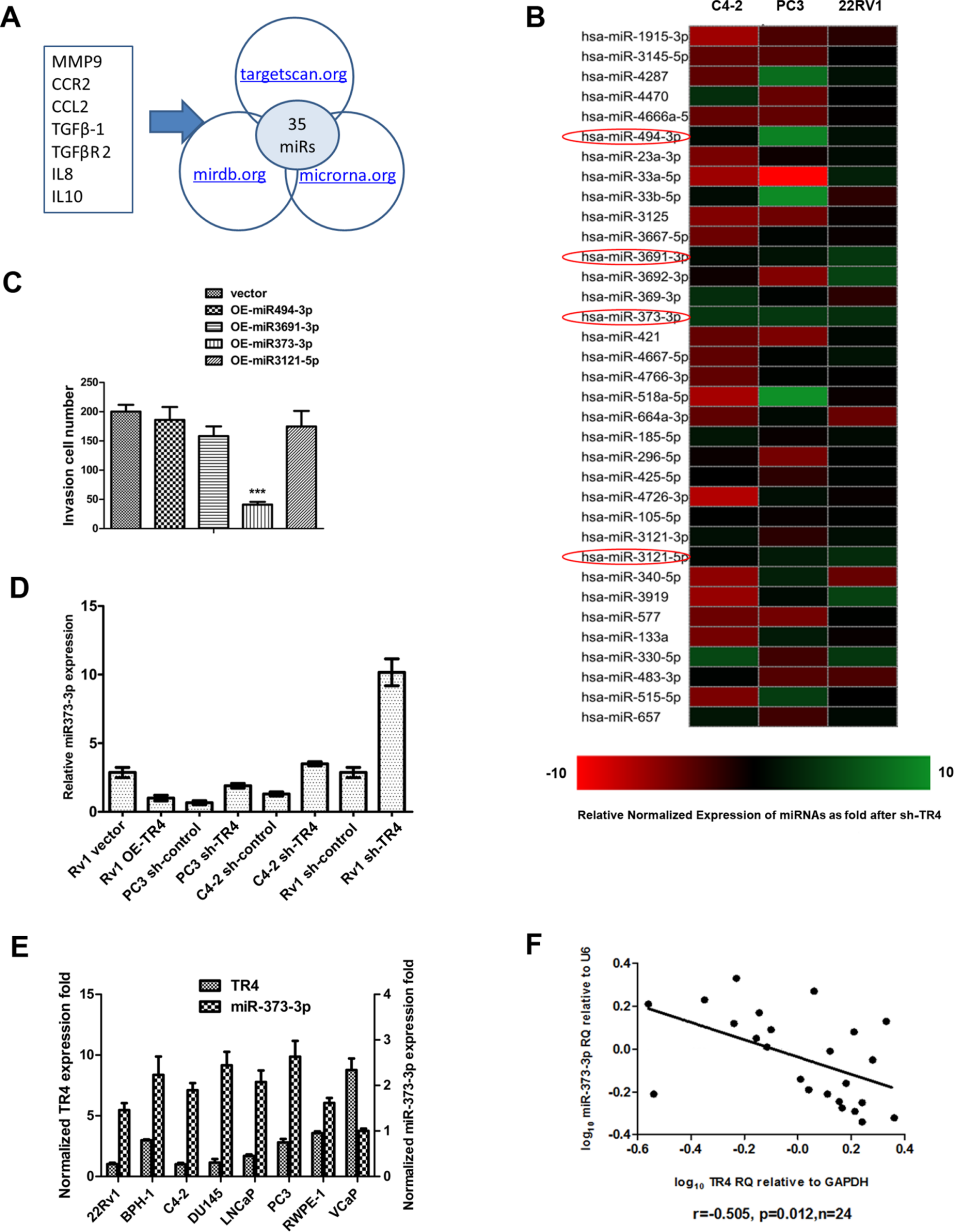
TR4 modulates miR-373-3p expression **A.** Some genes related to TR4 or PCa metastasis from published articles (20, 22–25) were screened for microRNA. These genes included MMP9, CCR2, CCL2, TGFβ-1, TGFβR2, IL8 and IL10. We picked out 35 overlapping miRNAs that target at least 3 of these putative genes from 3 microRNA prediction databases, targetscan.org, mirdb.org and microrna.org. **B.** Real-time PCR of 35 miRNAs related to PCa metastasis screened for si-TR4 in C4–2, PC3, and CWR22Rv1 cells. Relative normalized expression of miRNAs after knocking down TR4 was shown by different colors. When more green, the gene has a more negative correlation, the more red, the gene has a more positive correlation. There are 4 miRNAs up-regulated by TR4-siRNA in all 3 cell lines. **C.** We overexpressed (OE) the above miRNAs in the C4–2 cells with lentivirus transfection and found only miR-373-3p could suppress invasion (***P < 0.001). **D.** Real-time PCR (quantification) of miR-373-3p relative to U6 vector expression for shTR4 in C4–2, PC3, CWR22Rv1 (Rv1) cells and overexpressed TR4 in CWR22Rv1 (Rv1) cells. **E.** Comparison of real-time PCR expression (quantification) between miR-373-3p relative to U6 vector and TR4 relative to GAPDH in 8 human prostate cell lines. **F.** Plot of fold change of miR-373-3p related to fold change of TR4 in 24 PCa samples; RQ = relative quantity, *r* = −0.505, Pearson correlation coefficient.

To dissect the potential mechanism(s) by which TR4 can increase PCa cell invasion, we examined if TR4 might function through modulation of the miRNAs to increase PCa cell invasion as recently accumulating evidences [18] suggested that some selective miRNAs might be able to alter PCa metastasis. We first applied the bioinformatic approaches to determine the potential miRNAs that are predicted to be related to 7 metastasis-related genes, including MMP9, CCR2, CCL2, TGFβ-1, TGFβR2, IL8, and IL10 [18, 20–23]. From analysis of 3 different databases, including the Targetscan, miRDB and miRanda [24–26], we found 35 miRNAs that could target at least three of these 7 metastasis-related genes (Figure 2A). Then we applied the qPCR assay to validate the influence of these 35 predicted miRNAs by targeting the TR4 with TR4-siRNA in C4–2, PC3 and CWR22Rv1 cells and results revealed that 4 miRNAs (miR-494-3p, miR-3691-3p, miR-373-3p and miR-3121-5p) were up-regulated in all 3 cell lines (Figure 2B). We then applied an opposite approach using overexpressed (OE) miRNAs in the C4–2 cells and found only miR-373-3p could suppress PCa cell invasion (Figure 2C), and knocking-down TR4 increased miR-373-3p expression in all 3 PCa cell lines (Figure 2D).

Importantly, we also found miR-373-3p expression was negatively correlated with TR4 expression with higher expression of miR-373-3p vs lower expression of TR4 in 6 different PCa cell lines (C4–2, PC3, CWR22Rv1 (22Rv1), VCaP, LNCaP and DU145) plus two normal prostate cell lines (RWPE-1 and BPH-1) (Figure 2E). Furthermore, we also examined the TR4 vs miR-373-3p expression in human PCa tissues, and found a significant negative correlation (*r* = − 0.505, *p* = 0.012) between the TR4 expression vs miR-373-3p expression in 24 PCa specimens (Figure 2F).

Together, results from Figure 2A–2F suggest that TR4 can negatively regulate miR-373-3p expression in the PCa cell lines and tissues.

### miR-373-3p decreases PCa cell invasion

**Figure 3:**
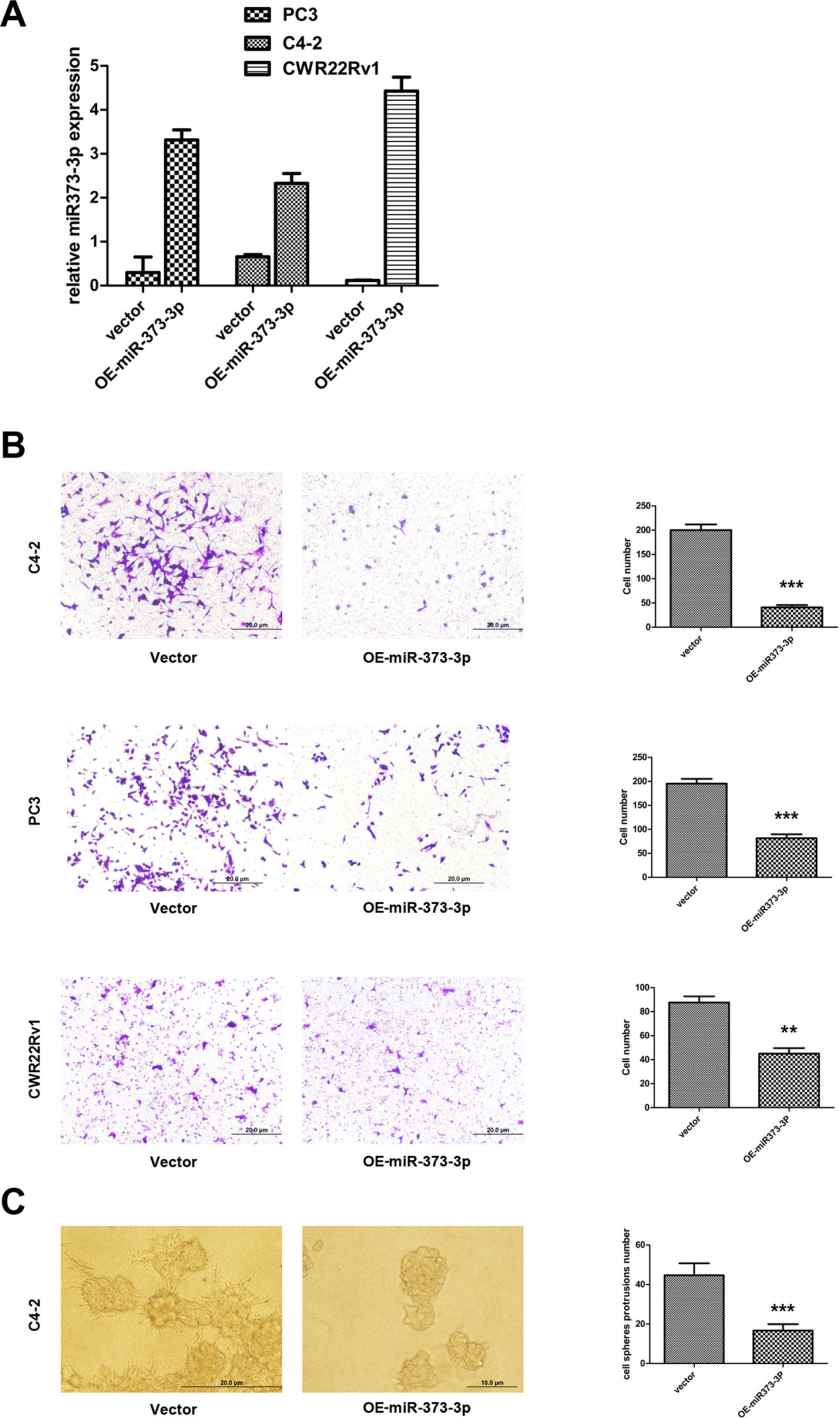
Effect of miR-373-3p on PCa cell invasion **A–C.** After stable lentivirus transfection, cells were seeded in a cell invasion chamber with the inner well coated with Matrigel and incubated for 24–36 h. **A.** Real-time PCR test expression (quantification) of miR-373-3p relative to U6 vector for stable overexpression (OE) of miR-373-3p in C4–2, PC3, and CWR22Rv1 cells. **B.** Invasion assay was performed in C4–2, PC3, CWR22Rv1 cells transfected with OE miR-373-3p or vector. Left: Representative microphotographs of invaded cells (magnification, 100 ×). Right: Quantitative analysis (cell numbers were counted in six randomly chosen microscopic fields per membrane). **C.** 3D spheroid invasion assay was performed in C4–2 cells transfected with OE-miR-373-3p or vector. Left: Representative microphotographs of cells (magnification, 200 ×). Right: Quantitative analysis (cell spheroid protrusions numbers were counted in six randomly chosen microscopic fields per membrane).*** *P* < 0.001, ** *P* < 0.01.

To examine the miR-373-3p effects on PCa cell invasion, we first stably transfected miR-373-3p using the lentivirus system and confirmed its expression in C4–2, PC3 and CWR22Rv1 cells (Figure 3A). We then performed invasion assay and found miR-373-3p could significantly suppress the PCa cell invasion in the 3 cell lines (Figure 3B).

Using another 3D invasion assay, we also confirmed that addition of miR-373-3p in C4–2 cells decreased cell invasion (Figure 3C).

Together, results from Figure 3A–3C suggest miR-373-3p can decrease PCa cell invasion.

### TR4 increases PCa cell invasion *via* suppression of miR-373-3p

**Figure 4:**
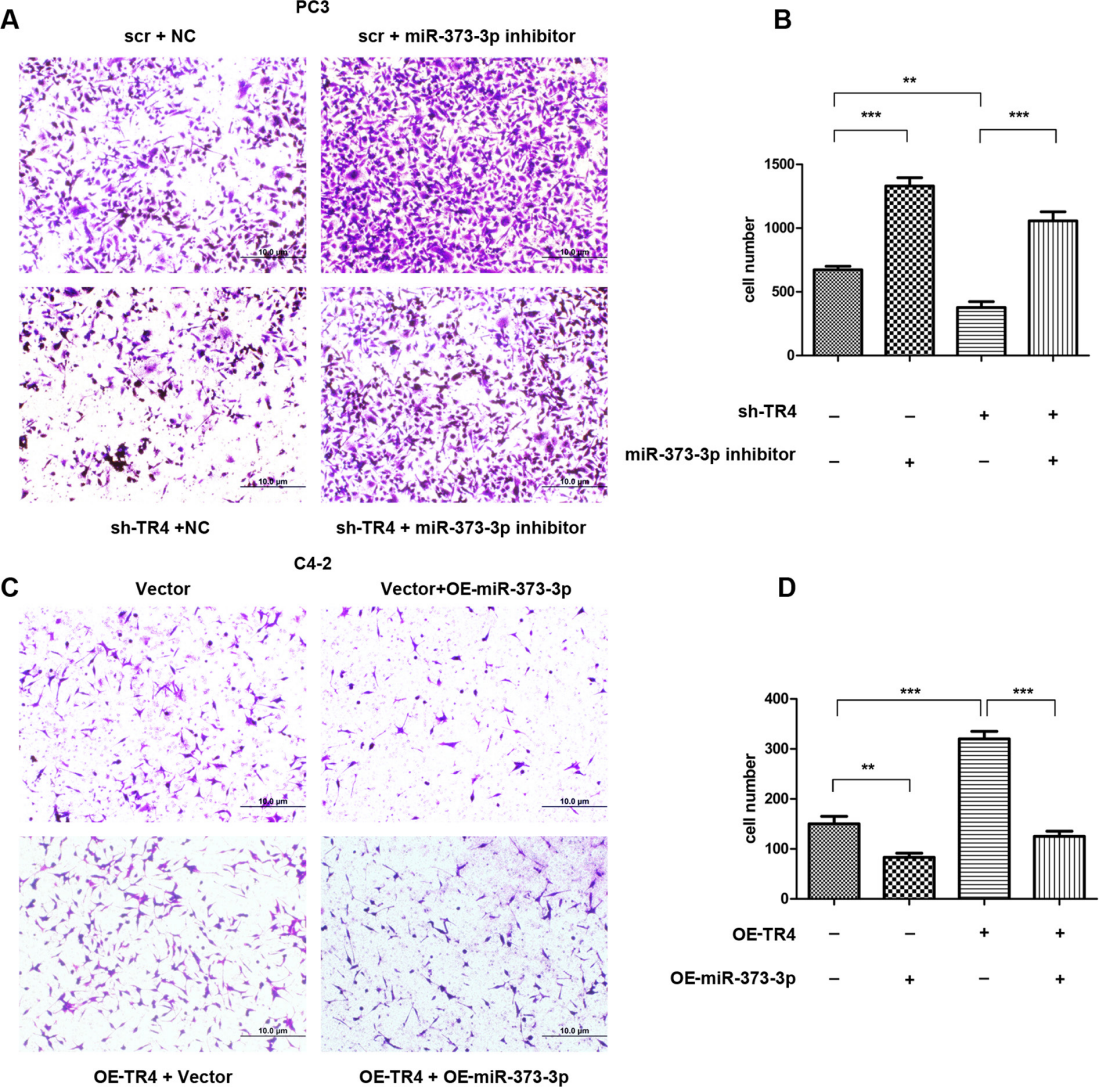
miR373-3p partially reverses TR4 function After 24 h lentivirus transfection, plasmid transient transfection by lipofectamine was performed. After 48 h, cells were seeded in cell invasion chambers with the inner wells coated with matrigel and incubated for 24–36 h. **A.** Invasion assay was performed in PC3 cells treated in 4 ways, scramble (scr) + negative control (NC), sh-TR4 + negative control, scr + miR-373-3p inhibitor and sh-TR4 + miR-373-3p inhibitor. Representative microphotographs of invaded cells (magnification, 100 ×). **B.** Quantitative analysis for the 4 PC3 groups (cell numbers were counted in six randomly chosen microscopic fields per membrane). **C.** Invasion assay was performed in C4–2 cells treated in 4 ways, vector, vector + OE-miR-373-3p, OE-TR4 + vector, and OE-TR4 + OE-miR-373-3p. Representative microphotographs of invaded cells (magnification, 100 ×). **D.** Quantitative analysis for the 4 C4–2 groups (cell numbers were counted in six randomly chosen microscopic fields per membrane).*** *P* < 0.001, ** *P* < 0.01.

To investigate whether miR-373-3p is involved in TR4-increased PCa cell invasion, we applied interruption approaches to see if targeting miR-373-3p might alter the TR4-increased PCa cell invasion. The results revealed that addition of miR-373-3p inhibitor increased PCa PC3 cell invasion (Figure 4A, upper right vs upper left and Figure 4B, lane 2 vs 1). Furthermore, knocking-down TR4 (sh-TR4) decreased PCa cell invasion (Figure 4A, lower left vs upper left and Figure 4B, lane 3 vs 1) and addition of miR-373-3p inhibitor partially reversed the sh-TR4 decreased PCa cell invasion (Figure 4A, lower right vs lower left and Figure 4B, lane 4 vs 3).

We then applied the opposite approach in C4–2 cells to confirm the above findings. The results revealed that addition of miR-373-3p decreased C4–2 cell invasion (Figure 4C, upper right vs upper left and Figure 4D, lane 2 vs 1). Furthermore, addition of functional TR4 increased C4–2 cell invasion (Figure 4C, lower left vs upper left and Figure 4D, lane 3 vs 1) that could be reversed after adding miR-373-3p in C4–2 cells (Figure 4A, lower right vs lower left and Figure 4D, lane 4 vs 3).

Together, results from Figure 4A–4D suggest that TR4 may be able to function through inhibiting the miR-373-3p expression to increase PCa cell invasion.

### TR4 regulates the TGFβR2/p-Smad3 signals *via* decreasing the miR-373-3p expression

**Figure 5:**
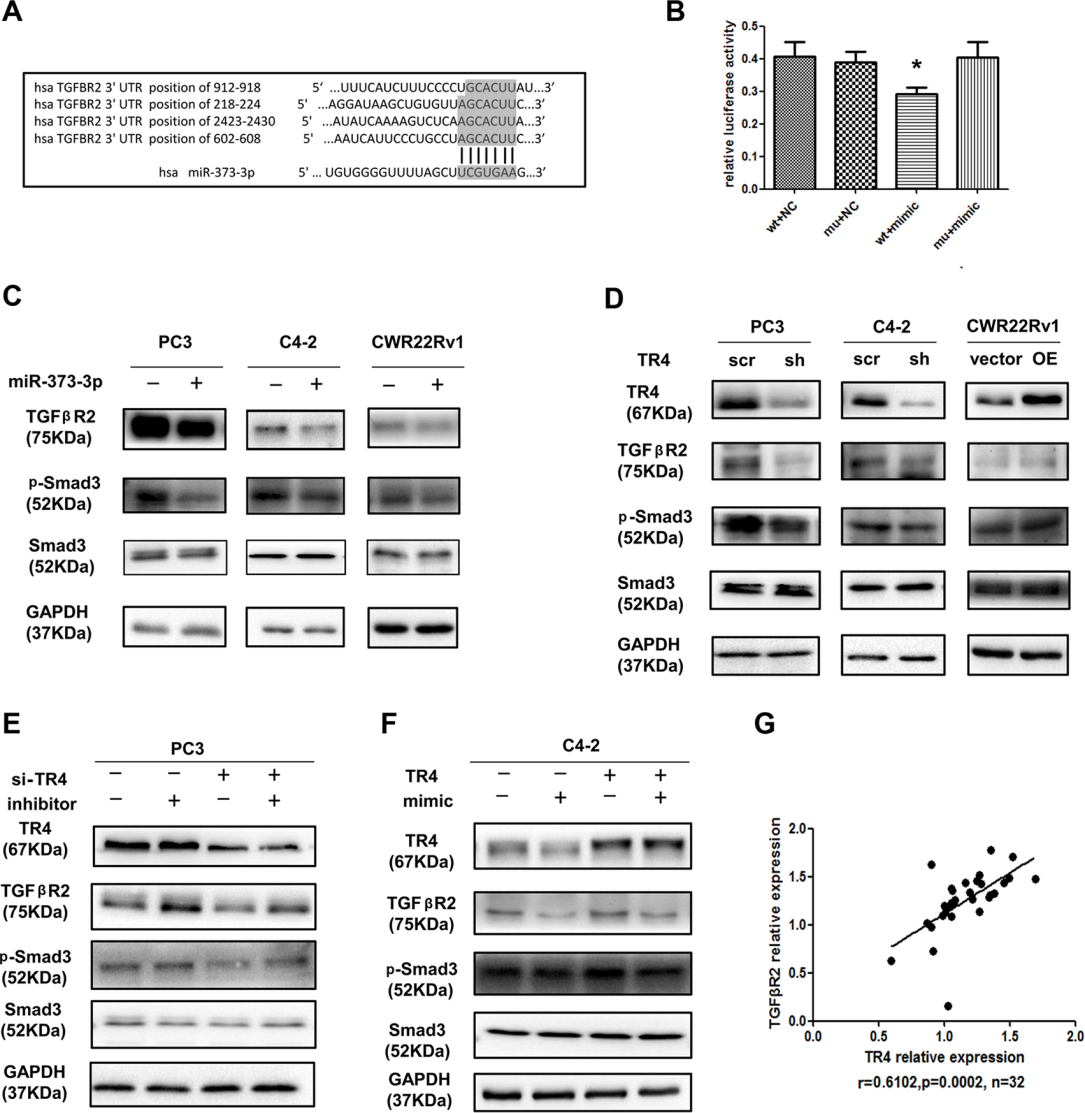
TR4 regulates the TGFβR2/p-Smad3 signals pathway *via* miR-373-3p **A.** Predicted duplex formation between human TGFβR2 3′ UTR and human miR-373-3p. There might be 4 target positions for miR-373-3p in TGFβR2 3′ UTR. **B.** Luciferase assay was performed with four groups; 1) psiCHECK-2-TGFβR2-wt_3′ UTR, 2) psiCHECK-2-TGFβR2-m3_3′ UTR, 3) TGFβR2-wt_3′ UTR + miR-373-3p mimic and 4) TGFβR2-m3_3′ UTR + miR-373-3p mimic. **C.** Western blot analysis for TGFβR2, p-Smad3 of total lysates of PC3, C4–2, CWR22Rv1 cells with vector (–) or overexpressed (+) miR-373-3p lentivirus transfection for 72–96 h. GAPDH was used to determine equal loading. **D.** Western blot analysis for TR4, TGFβR2, p-Smad3 of total lysates of PC3, C4–2, CWR22Rv1 cells treated with scramble (scr) and knocked down TR4 (sh) or vector and overexpressed (OE) TR4 lentivirus transfection for 72–96 h. GAPDH was used to determine equal loading. **E.** After 24 h lentivirus transfection of sh-TR4 and plko in PCa cells, miR-373-3p inhibitor transient transfection by lipofectamine was performed. After 48–72 h, protein was extracted for Western blot analysis. GAPDH was used to determine equal loading. **F.** After 24 h lentivirus transfection of overexpressed TR4 and vector in C4–2 and CWR22Rv1 cells, miR-373-3p mimic transient transfection by lipofectamine was performed. After 48–72 h, protein was extracted to do Western blot analysis. GAPDH was used to determine equal loading. **G.** Using NCBI GEO databases to analyze the PCa sample array (GEO dataset accession GSE35988), this data shows that TR4 and TGFβR2 expression in 32 metastasis PCa samples are positively correlated. *P* = 0.0002, *r* = 0.6102. Pearson correlation coefficient.

To further dissect the molecular mechanism(s) how miR-373-3p functions as a tumor suppressor to mediate the TR4-increased PCa cell invasion, we analyzed the miRNA prediction databases and found the linkage of miR-373-3p to TGFβR2 signals showing 4 potential positions to be targeted by miR-373-3p (Figure 5A). We then applied the luciferase assay to verify, and results revealed that addition of miR-373-3p mimic decreased the luciferase activity of TGFβR2–3′ UTR that is linked to the luciferase (Figure 5B). We also applied the western blot to confirm the protein expression and found addition of miR-373-3p decreased the expression of TGFβR2 and its downstream p-Smad3 in PC3, C4–2 and CWR22Rv1 cells (Figure 5C).

To link the miR-373-3p-suppressed TGFβR2/p-Smad3 to TR4, we knocked down TR4 with sh-TR4 and results revealed that decreased TR4 suppressed the expression of TGFβR2 and p-Smad3 at the protein level in PC3 and C4–2 cells, and overexpressing TR4 in CWR22Rv1 increased the expression of TGFβR2 and p-Smad3 at the protein level (Figure 5D). Importantly, we found the sh-TR4-suppressed TGFβR2 and p-Smad3 expressions (Figure 5E, lane 3 vs 1) could be significantly reversed after addition of miR-373-3p inhibitor in PC3 cells (Figure 5E, lane 4 vs 3). As expected, TR4 enhanced TGFβR2 and p-Smad3 expression (Figure 5F, lane 3 vs 1) could be partially reversed after addition of miR-373-3p mimic in C4–2 cells (Figure 5F, lane 4 vs 3).

Using NCBI GEO databases to analyze the PCa sample array (GEO dataset accession GSE35988) [19], we also found the positive correlation (*r* = 0.6102, *p* = 0.0002) of TR4 and TGFβR2 in 32 metastatic PCa samples (Figure 5G).

Together, results from Figure 5A–G demonstrated that TR4 might function through inhibiting miR-373-3p to regulate TGFβR2/p-Smad3 signals to increase PCa cell invasion.

### TR4 increases PCa metastasis *via* suppression miR-373-3p-TGFβR2 signals *in vivo*

**Figure 6:**
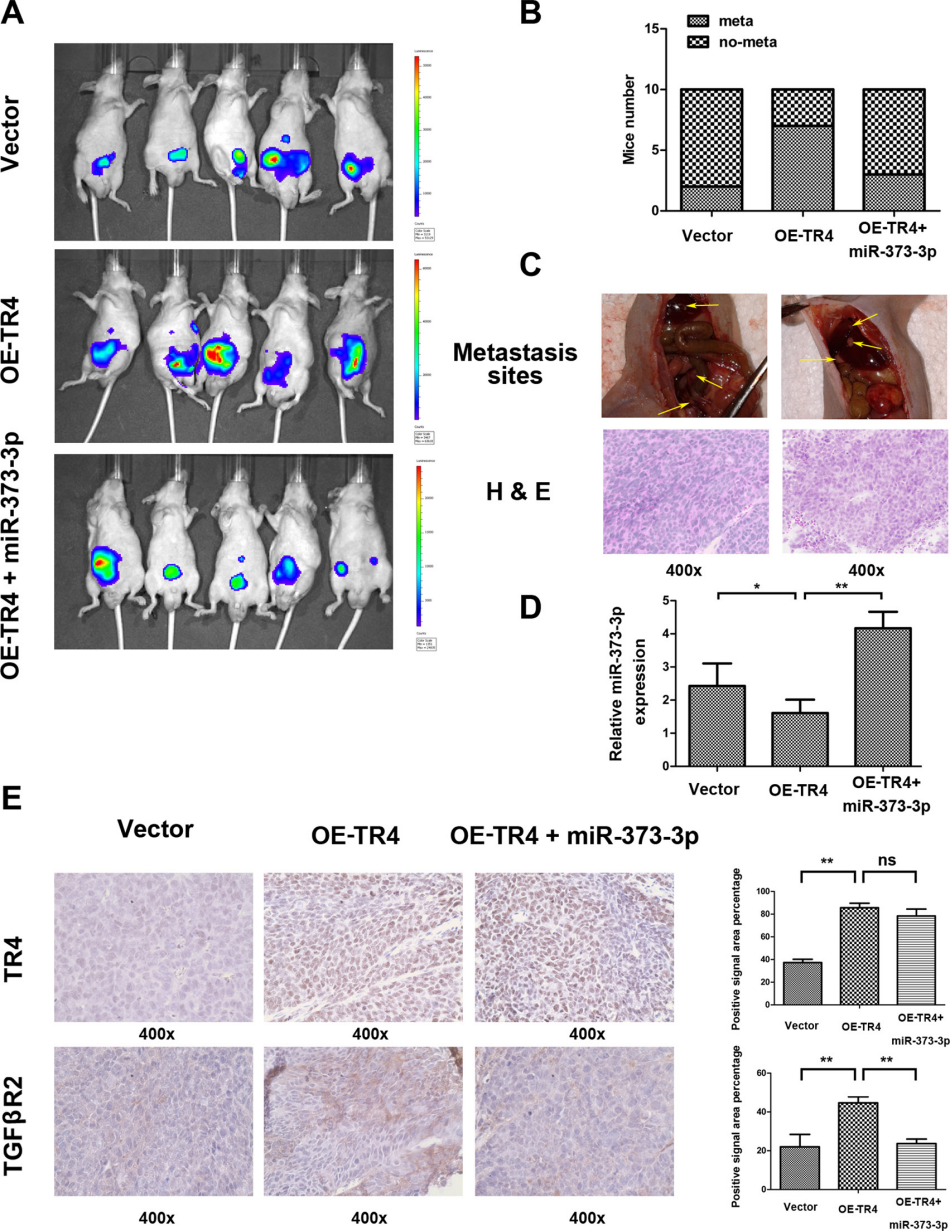
TR4 promotes metastasis of PCa *via* miR-373-3p *in vivo* The CWR22Rv1 cells transduced with vector, overexpressed (OE)-TR4, or OE-TR4 + miR-373-3p were orthotopically implanted into the anterior prostates of nude mice. After 6 weeks implantation, the PCa growth and metastasis were monitored by IVIS images, following tail vein injection of Luciferin, and the mice were then sacrificed. Then H&E and IHC tissue staining confirmed the expression of genes. **A.** Representative IVIS images six weeks after implanting CWR22Rv1 cells into the anterior prostates of nude mice. **B.** Incidence of metastases in different groups of mice at six weeks after cells implantation. **C.** Representative images of mouse with pelvic lymph node metastasis and with diaphragm metastasis (upper panels) and H&E staining (lower panels) of metastatic foci are shown. **D.** RT-QPCR assay detecting miR-373-3p expression in 3 groups. *p < 0.05, **p < 0.01. **E.** IHC staining for detecting TR4 (upper panels) and TGFβR2 (lower panels) expressions in tumor tissues obtained from 3 groups mice. Quantification at right. ** *P* < 0.01, ns no significance.

To further confirm the above *in vitro* data in an *in vivo* mouse model, we first stably transfected CWR22Rv1 cells with firefly luciferase reporter gene (Luc-CWR22Rc1) and prepared cell lines transduced with vector, OE-TR4, and OE-TR4 + OE-miR-373-3p, and orthotopically xenografted cells (10^6^) into anterior prostates of 10 mice/group. After 6 weeks IVIS images revealed that the mice with overexpressed TR4 cells had more metastatic foci than mice injected with vector cells (Figure 6A–6B). Importantly, injecting mice with cells overexpressing both miR-373-3p and TR4 then reversed the increased meastatic foci formation (Figure 6A–6B).

H&E staining of those metastatic foci confirmed that the metastatic foci found in diaphragm, pelvic wall and hepatic region were indeed PCa (Figure 6C). RT-qPCR assay confirmed that miR-373-3p expression in mice with overexpressed TR4 was lower and in mice with overexpressed TR4 plus miR-373-3p was higher (Figure 6D). IHC staining for TR4 and TGFβR2 were also in agreement with *in vitro* results (Figure 6E).

Together, results from Figure 6A–6E *in vivo* mouse model studies are in agreement with the above *in vitro* cell lines studies and demonstrated that targeting TR4 could suppress PCa cell invasion *via* regulation of miR-373-3p/TGFβR2/p-Smad3 signals.

## DISCUSSION

Although ADT is the standard treatment for advanced PCa, most patients eventually relapse with castration resistance [2–4]. Interestingly, recent studies also indicated some ADT might enhance PCa metastasis [27]. Among several signals that affect the PCa progression, we found that TR4 might play a protective role to suppress the prostate tumorigenesis, knocking-out TR4 might increase PIN and/or prostatic carcinoma formation [12], and targeting TR4 with lentiviral silencing might alter the chemo-resistance of PCa stem/progenitor cells [28]. In contrast, a recent study indicated that TR4 might play positive roles to increase PCa metastasis [18]. However, the detailed mechanisms, especially the linkage of miRNAs to TR4 positive roles to increase PCa metastasis, remain unclear. Here we demonstrated that TR4 could increase PCa cell invasion *via* inhibition of miR-373-3p, suggesting TR4 might play dual roles with a suppressor role in PCa initiation and a promoter role in metastasis development.

As a key player in PCa, TR4 may have multiple ways to either modulate or to be modulated by miRNAs. Here we screened 35 miRNAs and found some of them are regulated by TR4 in PCa. Interestingly, we found miR-373-3p might function as a suppressor to inhibit TR4. Early studies suggested that miR-373-3p might function as a potential novel oncogene in testicular germ-cell tumors [29] and breast cancer [30]. However, other studies reported that miR-373-3p could also function as a tumor suppressor in breast tumor [31] and pancreatic cancer [32]. These studies suggest that miR-373-3p has dual roles as both promoter and suppressor in different types of tumors.

Interestingly, early studies also suggested TGFβ signaling might play dual roles as a suppressor and a promoter to influence the tumor progression [33, 34]. The loss of TGFβR2 is associated with poor clinical prognosis and is a predictor of poor prognosis in early stage breast cancer but overexpression of the TGFβ ligand is associated with the metastatic phenotype in many tumors [35, 36], and Davies et al. reported that TGFβ enhanced metastasis in Ras-transfected human malignant epidermal keratinocytes [37].

Our previous study found that TR4 could promote PCa metastasis *via* modulation of the CCL2/CCR2 signals, a key player of EMT [18]. In this study we further found that TR4 could increase PCa cells invasion *via* inhibiting miR-373-3p and consequently enhance the TGFβR2/p-Smad3 signals. These results suggest that TR4 may increase PCa metastasis through multiple mechanisms: not only the regular ligands/receptors, but also intercellular miRNAs/mRNAs. Similar phenomena also occurred in p53 signals showing p53 could modulate Bcl2 at the transcriptional level to regulate cell apoptosis, and at the same time p53 could also function through the miR34 family to regulate the apoptosis [38].

Our results showing TR4 might function as suppressor through modulation of miR-373-3p to alter the expression of TGFβR2/p-Smad3 to increase the PCa metastasis, therefore represents the 3rd molecule (in addition to miR-373-3p and TGFβ) in this newly identified pathway that has both suppressor and stimulator roles to influence the tumor metastasis. These complicated signals found in PCa suggest that the PCa metastasis process may be very hard to inhibit and targeting any particular molecule might be only effective in some and not all conditions.

To summarize, the studies presented herein demonstrate that TR4 might be able to function through inhibiting the miR-373-3p expression to alter the TGFβR2/p-Smad3 signals to increase the PCa cell invasion. Targeting TR4, miR-373-3p or TGFβR2/p-Smad3 may become a new potential therapeutic approach to better suppress PCa metastasis.

## MATERIALS AND METHODS

### Cell culture

Human PCa cell lines C4–2, CWR22Rv-1, PC3, DU145, VCaP, and LNCaP, and human benign prostate cell lines RWPE-1 and BPH-1 were obtained from the American Type Culture collection (ATCC, Rockville, MD). RWPE-1 cells were maintained in complete keratinocyte serum-free media (KSF-M), supplemented with 1% penicillin/streptomycin/amphoterycin B, 50 mg/ml bovine pituitary extract and 5 ng/ml epidermal growth factor (Life Technologies, Barcelona, Spain). C4–2, CWR22Rv-1, VCaP, LNCaP, and BPH-1 cells were cultured in RPMI-1640 media containing 1% penicillin and streptomycin, supplemented with 10% fetal bovine serum (FBS). PC3 and DU145 cells were cultured in DMEM media containing 1% penicillin and streptomycin, supplemented with 10% FBS. All cell lines were cultured in a 5% (v/v) CO_2_ humidified incubator at 37°C.

### Construct/generate PCa cell lines with differential stable expression of TR4 and/or miR-373-3p

The miRNA-373-3p expression plasmid was generated by cloning the genomic pre–miR-373-3p gene, with a 200-bp sequence on each flanking side, into retroviral transfer plasmid pWPI to generate plasmid pWPI-miR-373-3p, as in the previous report [27]. To generate overexpressed TR4, overexpressed miR-373-3p and TR4 knocked-down stable clones, C4–2, CWR22Rv-1, and PC3 cells were transfected with lentiviral vectors, pWPI-TR4/pWPI-Vec, pWPI-miR-373-3p/pWPI-Vec or pLKO1.0-TR4-si/pLKO1-scr, the psPAX2 packaging plasmid, and pMD2.G envelope plasmid, then transfected into 293T cells using the standard calcium phosphate transfection method for 48 hrs to obtain the lentivirus soup that was collected and frozen in −80°C for later use. The cells were transfected using the LipofectAMINE 3000 (Invitrogen) reverse transfection protocol, according to the manufacturer's instructions. The miR-373-3p mimic (10 nM), miR-373-3p inhibitor (10 nM) and negative control (NC) were from Qiagen (Valencia, CA).

### Quantitative real-time PCR

For RNA extraction, total RNAs were isolated using Trizol reagent (Invitrogen, Grand Island, NY). 1–2 μg of total RNA was subjected to reverse transcription using Superscript III transcriptase (Invitrogen). Quantitative real-time PCR (qRT-PCR) was conducted using a Bio-Rad CFX96 system with SYBR green to determine the mRNA expression level of a gene of interest. Expression levels were normalized to the expression of GAPDH mRNA. The primers for TR4 are: Forward: 5′-TCC CCA CGC ATC CAG ATA ATC-3′; and Reverse: 5′-GAT GTG AAA ACA CTC AAT GGG C-3′. The primers for GAPDH are: Forward: 5′-GGA GCG AGA TCC CTC CAA AAT-3′; and Reverse: 5′-GGC TGT TGT CAT ACT TCT CAT GG-3′. The miRNAs were isolated using PureLink^®^miRNA kit. In brief, 50 ng small RNAs were processed for poly A addition by adding 1 unit of polymerase with 1 mM ATP in 1 × RT buffer at 37°C for 10 mins in 10 μl volume, and then heat inactivated at 95°C for 2 mins, then add 50 pmol anchor primer up to 12.5 μl and incubate at 65°C for 5 mins. For the last step of cDNA synthesis, add 2 μl 5 × RT buffer, 2 μl 10 mM dNTP, 1 μl reverse transcriptase up to total of 20 μl and incubate at 42°C for 1 hr. Quantitative real-time PCR was conducted using a Bio-Rad CFX96 system with FAM/FITC to determine the miRNA expression level. Expression levels were normalized to the expression of U6 RNA.

### Western blot analysis

Cells were lysed in RIPA buffer and proteins (20–50 μg) were separated on 8–10% SDS/PAGE gel and then transferred onto PVDF membranes (Millipore, Billerica, MA). After blocking membranes, they were incubated with appropriate dilutions of specific primary antibodies against GAPDH (Santa Cruz, #sc-166574, Paso Robles, CA), TR4 (Perseus Proteomics, #PP-H0107B-00), TGFβR2 (Abcam, #ab17650, San Deigo, CA), p-Smad3 (Ser425, Santa Cruz, #sc-11769) or Smad3 (Santa Cruz, #sc-101154). The blots were incubated with HRP-conjugated secondary antibodies and visualized using ECL system (Thermo Fisher Scientific, Rochester, NY).

### Invasion assay

The invasion capabilities of PCa cells were determined by the transwell assays. To prepare chambers for assay, 10 mL of Matrigel (BD, Inc) was dissolved in 50 mL serum-free DMEM or RPMI-1640, and 100 μl mixed Matrigel added to the upper chambers of transwells containing 8 μm-pore-size polycarbonate membrane filters (Corning, Inc., Corning, NY), and put into the incubator for 5 hrs. PCa cells were then harvested and seeded with serum-free DMEM or RPMI-1640 media into the upper chamber at 1 × 10^5^ cells/well, and the bottom chambers contained DMEM or RPMI-1640 with 10% FBS, and then transwells were incubated for 48 hrs at 37°C. Following incubation, the invaded cells attached to the lower surface of the membrane were fixed using 4% paraformaldehyde and stained with 1% toluidine blue. Cell numbers were counted in six randomly chosen microscopic fields (100 ×) per membrane.

### 3D invasion assay

The 3D invasion assay was modified from the previous report [39]. Briefly, 1 × 10^4^ of cells in 200 μl media containing 1% Matrigel was plated into the collagen/Matrigel mixture coated 24-well plates. The media was replenished every 3 days for 2 wks and the spheres with/without protrusions recorded under microscope. 100 spheres were recorded in each well to determine the ratio of cells with/without protrusions.

### Luciferase reporter gene assays

1 × 10^3^ of cells/well in 24-well plates were transfected with psiCHECK-2-TGFβR2-wt_3′ UTR (Addgene, Plasmid 31882), psiCHECK-2-TGFβR2-m3_3′ UTR (Addgene, Plasmid 31883), TGFβR2-wt_3′ UTR + miR-373-3p mimic (QIAGEN, Lot no:171418189) and TGFβR2-wt_3′ UTR + miR-373-3p mimic using Lipofectamine 3000 (Invitrogen). After 48 hrs transfection, cell lysates were prepared with Passive Lysis Buffer (Promega, Madison, WI) and luciferase activities were measured using the Dual Luciferase Reporter Assay (Promega, Madison, WI).

### *In vivo* metastasis studies

Male 6–8 weeks old athymic nude mice were purchased from NCI. 30 mice were divided into 3 groups. 1 × 10^6^ cells (mixture with Matrigel, 1:1) were injected into the anterior prostate of all mice. Group 1 mice were injected with Luc-CWR22Rv1 cells transduced with vector, Group 2 mice with Luc-CWR22Rv1 cells transduced with overexpressed TR4, and Group 3 mice with Luc-CWR22Rv1 cells transduced with overexpressed TR4 and overexpressed miR-373-3p. Every week, tumor growth/metastasis was monitored by *in vivo* imaging system (IVIS Spectrum, Caliper Life Sciences, Hopkinton, MA) following tail vein injection of Luciferin. At the end of treatment (6 weeks), mice were sacrificed and tumor growth and metastases at lymph nodes as well as to distant organs were analyzed and examined by H&E staining.

### H&E and immunohistochemical (IHC) staining

Tissues were fixed in 10% (v/v) formaldehyde in PBS, embedded in paraffin, and cut into 4 μm sections and used for H&E staining and IHC staining with human TR4/TGFβR2 antibodies. To enhance antigen exposure, the slides were treated with EDTA at 98°C for 10 min for antigen retrieval. The slides were incubated with endogenous peroxidase blocking solution to inhibit endogenous peroxidase, and then were incubated with the primary antibody at room temperature for 60 min. After rinsing with Tris-buffered saline, the slides were incubated for 45 min with biotin-conjugated secondary antibody, washed, and then incubated with enzyme conjugate horseradish peroxidase (HRP)-streptavidin. Freshly prepared DAB (Zymed, South San Francisco, CA) was used as the substrate to detect HRP. Finally, slides were counter-stained with hematoxylin and mounted with aqueous mounting media.

### Statistical analysis

Data are expressed as mean ± SEM from at least 3 independent experiments. Statistical analyses involved paired *t*-test with SPSS 17.0 (SPSS Inc., Chicago, IL). For the *in vivo* studies, measurements of tumor metastasis among the three groups were analyzed through one-way ANOVA coupled with the Newman-Keuls test. *p* < 0.05 was considered statistically significant.
